# A prospective pilot study of genome-wide exome and transcriptome profiling in patients with small cell lung cancer progressing after first-line therapy

**DOI:** 10.1371/journal.pone.0179170

**Published:** 2017-06-06

**Authors:** Glen J. Weiss, Sara A. Byron, Jessica Aldrich, Ashish Sangal, Heather Barilla, Jeffrey A. Kiefer, John D. Carpten, David W. Craig, Timothy G. Whitsett

**Affiliations:** 1 Western Regional Medical Center, Cancer Treatment Centers of America, Goodyear, Arizona, United States of America; 2 Translational Genomics Research Institute, Phoenix, Arizona, United States of America; Universita Campus Bio-Medico di Roma, ITALY

## Abstract

**Background:**

Small cell lung cancer (SCLC) that has progressed after first-line therapy is an aggressive disease with few effective therapeutic strategies. In this prospective study, we employed next-generation sequencing (NGS) to identify therapeutically actionable alterations to guide treatment for advanced SCLC patients.

**Methods:**

Twelve patients with SCLC were enrolled after failing platinum-based chemotherapy. Following informed consent, genome-wide exome and RNA-sequencing was performed in a CLIA-certified, CAP-accredited environment. Actionable targets were identified and therapeutic recommendations made from a pharmacopeia of FDA-approved drugs. Clinical response to genomically-guided treatment was evaluated by Response Evaluation Criteria in Solid Tumors (RECIST) 1.1.

**Results:**

The study completed its accrual goal of 12 evaluable patients. The minimum tumor content for successful NGS was 20%, with a median turnaround time from sample collection to genomics-based treatment recommendation of 27 days. At least two clinically actionable targets were identified in each patient, and six patients (50%) received treatment identified by NGS. Two had partial responses by RECIST 1.1 on a clinical trial involving a PD-1 inhibitor + irinotecan (indicated by *MLH1* alteration). The remaining patients had clinical deterioration before NGS recommended therapy could be initiated.

**Conclusions:**

Comprehensive genomic profiling using NGS identified clinically-actionable alterations in SCLC patients who progressed on initial therapy. Recommended PD-1 therapy generated partial responses in two patients. Earlier access to NGS guided therapy, along with improved understanding of those SCLC patients likely to respond to immune-based therapies, should help to extend survival in these cases with poor outcomes.

## Introduction

Lung cancer is the leading cause of cancer deaths in both women and men in the United States and throughout the world. In 2017, it is expected that there will be 222,500 new cases of lung cancer with more than 155,000 deaths in the United States alone [1]. As small cell lung cancer (SCLC) accounts for between 13–15% of lung cancer, ~30,000 new cases will be diagnosed in the US in 2017 [2, 3]. Though initially responsive to front-line chemotherapy, the vast majority of SCLC will have disease progression (PD). For SCLC patients who progress after first-line therapy, the standard FDA-approved treatment options are either topotecan or the combination of cyclophosphamide, doxorubicin, and vincristine (CAV) [3, 4]. Response rates for PD are substantially lower, and have been reported as low as 7% for oral topotecan in cisplatin-refractory patients or 8–15% for CAV [5, 6]. In a phase III trial for second-line treatment for SCLC, amrubicin and topotecan had response rates of 31.1 and 16.9%, respectively, with no change in overall survival (7.5–7.8 months) or progression-free survival (3.5–4.1 months) [7]. SCLC, especially extensive-stage, that has progressed after 1^st^ line therapy has few effective treatments and no new class of approved therapies in decades [8].

While non-small cell lung cancers (NSCLC) harbor recurrent oncogenic mutations that can be targeted therapeutically (e.g. epidermal growth factor receptor (*EGFR*) or anaplastic lymphoma receptor tyrosine kinase (*ALK*)) [9], the most prevalent molecular alterations in SCLC are in *TP53*, *RB1*, and *MYC*, alterations without effective targeted therapies [10]. Another group recently interrogated 148 lung neuroendocrine tumors, including 33 SCLC, and demonstrated the prevalence of *TP53* and *RB1* while suggesting significant roles for chromatin-remodeling genes in lung neuroendocrine pathogenesis [11]. Alterations in PI3K signaling (especially *PIK3CA*) were suggested for therapeutic targeting in carcinomas, although the pathway was altered in a subset of tumors (11.7%). While novel, targeted therapeutics against NOTCH signaling, PI3K-PTEN pathway, and FGFR proteins have shown pre-clinical promise, survival rates for recurrent, extensive-stage SCLC remain dismal [12, 13]. More recently, immune checkpoint inhibitors have shown efficacy in SCLC with PD-1 inhibition via pembrolizumab demonstrating a response rates of 35% in a phase I/II trial of SCLC patients[14], while nivolumab had a response rate of 10% as a single agent or 19–23% in combination with ipilimumab in patients with relapsed SCLC [15]. Thus, a deeper understanding of the driver alterations in SCLC, especially in PD, and an understanding of those patients likely to respond to immune checkpoint blockade should improve patient outcome.

We know that somatic alterations (i.e., point mutations, small insertions and deletions, rearrangements, gains and losses) occur at the DNA level in cancer. These somatic events can drive tumorigenesis, metastatic progression, and/or drug resistance. More importantly, specific somatic alterations can be targeted by therapeutics. For the first time, technology now offers us the ability to survey the global somatic landscape of cancer: it is possible to sequence, analyze, and compare the matched tumor and normal genomes of an individual patient. The focus of the current study is to improve treatment strategies for individuals with SCLC, especially with PD. We recently completed a pilot study (NFCR trial) for patients with advanced rare cancers (NCT01443390) to evaluate the utility of identifying potential therapeutic targets in tumors from patients whose cancers had progressed on standard therapies. Our preliminary findings in advanced cancer using next-generation sequencing (NGS) indicate most tumors possess at least one actionable target for a conventional FDA-approved agent or a drug in clinical development [16]. Paired tumor-normal exome and transcriptome sequencing efficiency, coverage, cost, and analytics has improved over the last decade and is being applied in the clinic.

In this prospective study, we employed genome-wide exome and RNA-sequencing to identify genomic events and associated expression changes in advanced SCLC, and sought to prescribe systemic therapies based on the results.

## Materials and methods

### Study design

The rationale for this study is that NGS could be used to identify as many potentially actionable genomic abnormalities that could be targeted using available therapies in advanced SCLC patients. This single center, prospective, single-arm study was in patients with advanced SCLC that progressed on 1^st^ line standard systemic therapy. The study was conducted in accordance with the Declaration of Helsinki and was approved by the Western Institutional Review Board (WIRB^®^ Protocol #20132159) (clinicaltrials.gov NCT02297087) on September 15, 2014. Study enrollment was between October 2014 and April 2016 and last follow-up was completed in February 2017 (S1 File). The study was registered on clinicaltrials.gov within 21 days of enrollment of the first participant in compliance with FDAAA 801 requirements. The authors confirm that all ongoing and related trials for this drug/intervention are registered. Each participant signed informed consent before study related procedures were conducted. To participate, patients must have been age ≥18 and willing to undergo a fresh tumor biopsy. Other eligibility criteria included: Karnofsky performance status ≥70%, life expectancy >3 months, and baseline laboratory data indicating acceptable bone marrow reserve, liver, and renal function. Main exclusion criteria were symptomatic or untreated central nervous system (CNS) metastases, known active infections requiring intravenous antimicrobial therapy, pregnant or breast-feeding women, or tumor that was inaccessible for an adequate biopsy. Participation on another clinical trial involving treatment prior to or during participation on this study was allowed. Clinical annotation related to the tumor samples analyzed such as age at time of consent, gender, histology, stage at the time of consent, type of prior therapy, and duration and best response to those therapies were collected and summarized.

### Genome-wide exome and mRNA-sequencing analysis

Following patient consent, fresh frozen tumor tissue was acquired by clinical tumor biopsy for profiling analysis, and peripheral blood mononuclear cells collected for constitutional DNA analysis. Specimens were de-identified and coded, and shipped overnight to Ashion^®^ (http://www.ashion.com), a Clinical Laboratory Improvement Amendments (CLIA)-certified, College of American Pathologists (CAP)-accredited laboratory, for sample processing and analysis. A board-certified pathologist at Ashion reviewed a portion of each specimen to confirm adequate tumor content (>20% tumor). DNA and RNA were extracted, and genome-wide exome sequencing and RNA-sequencing was performed by Ashion^®^ using their GEM Genome Wide (GEM GW) exome sequencing and RNA-sequencing platforms. Tumor/normal GEM GW exome sequencing was used to provide clinical whole exome analysis for identification of somatic coding point mutations, small insertions, and deletions within exons for >20,000 genes, and for regional whole genome analysis to detect copy number changes and structural events. Tumor RNA-sequencing was performed for fusion detection. Comparison to commercially available normal lung RNA was used for differential expression. The average GEM GW exome target coverage was 420X (range, 222X-560X) for tumor samples and 201X (range, 118X-270X) for peripheral blood samples. For tumor samples, all samples had more than 95% of target bases covered with at least 20X coverage (average 98.1%, range 97.9–98.3%). More than 80% of target bases had at least 100X coverage (average 92.5%, range 81.7–95.9%). Tumor RNA-sequencing had an average of >248 million aligned reads (range, 170–377 million). Sequence alignment, variant calling, and variant filtering were performed using a custom bioinformatics pipeline, as previously described [17–19]. Data were aligned to build 37 of the human reference genome. Data are deposited to dbGaP under accession, phs001366.v1.p1. (https://www.ncbi.nlm.nih.gov/projects/gap/cgi-bin/study.cgi?study_id=phs001366.v1.p1).

### Genomics-guided treatment recommendations

Specific somatic alterations were matched to potential therapeutic options (within the study pharmacopeia) using custom drug rules, as previously described [18, 19]. The pharmacopeia for this study consisted of FDA-approved oncology drugs, including on-label and off-label use of these agents. An interpretive genomic report outlining the somatic alterations with potential therapeutic implications, as well as alterations in genes implicated in cancer, was generated for each patient. A molecular tumor board, consisting of the treating oncologist, clinical investigator, genomics experts and/or biology experts, reviewed the interpretive genomics report and discussed potential treatment options. A treatment strategy was recommended, and, if agreed upon by the treating oncologist and patient, the recommendation was pursued for treatment. If the prescription was denied by the insurer, appeal to insurance was made followed by request for drug from the manufacturer using a patient assistance or drug acquisition program.

### Study endpoints

The primary objective of this study was to launch a pilot study enrolling 12 eligible patients with advanced SCLC and to obtain the necessary tumor biopsies to yield sufficient DNA and RNA for genome-wide interrogation. The secondary objective was to provide a new clinical paradigm in the treatment of SCLC such that each individual patient would be treated with a single-agent or combination therapy of commercially available agents that relates to particular target(s) that have been identified via NGS.

## Results

### Patient characteristics

All patients were seen and evaluated for inclusion in this study between October 2014 and April 2016 at a single center.

**Fig 1:**
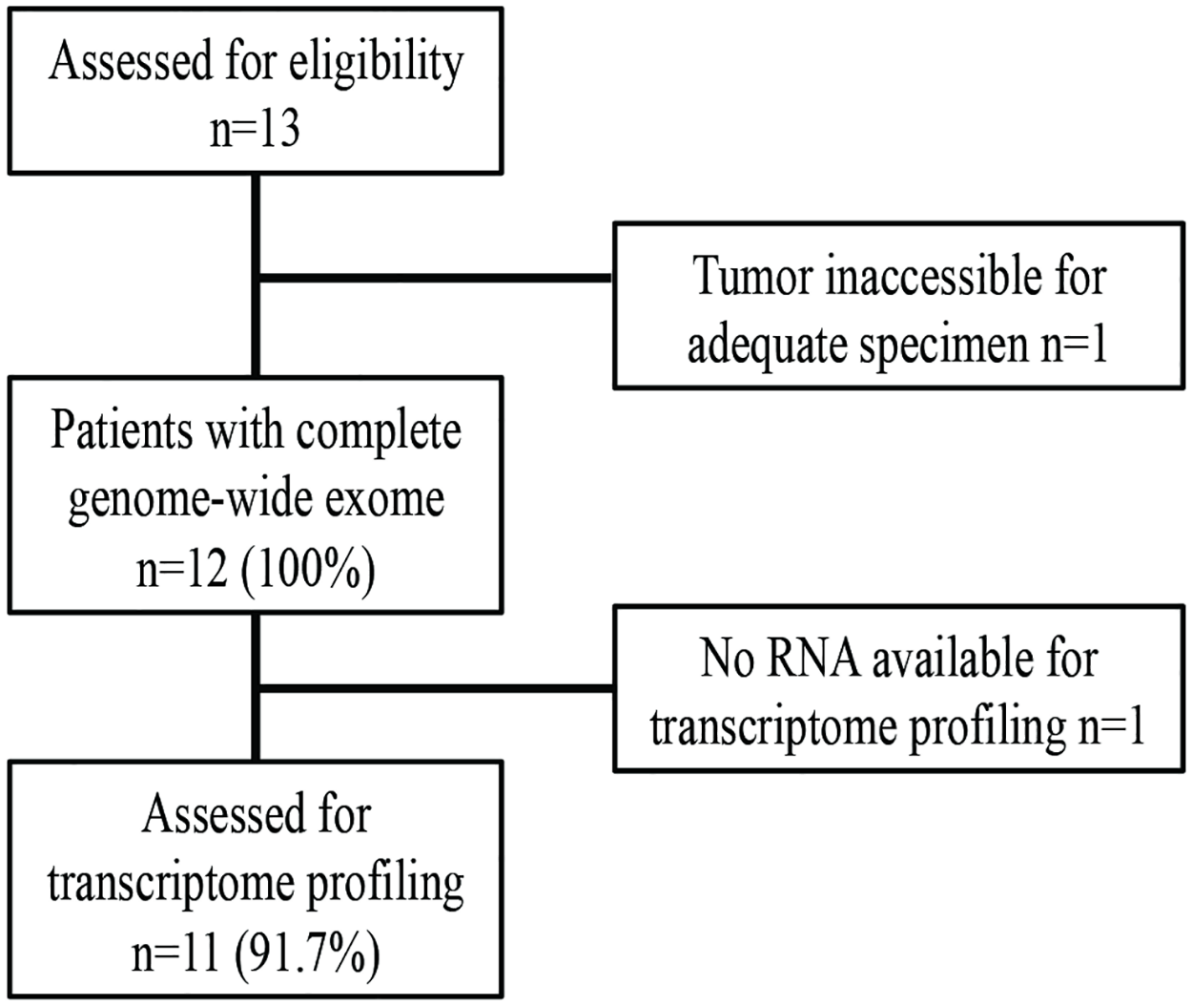
CONSORT diagram. Displays the flow of the 13 patients who consented and were evaluated for the study. There was one screen failure due to anticipated inadequate sample yield because of tumor location.

Fig 1 details the Consolidated Standards of Reporting Trials (CONSORT) diagram demonstrating the flow of the 13 patients who consented and were evaluated for the study (S2 File). There was one screen failure due to anticipated inadequate sample yield because of tumor location. The cohort included 10 women, median age was 56.5 years, and included 3 never smokers. All patients received prior platinum-based chemotherapy and were receiving >1^st^ line systemic treatment while awaiting NGS results.

For the 12 eligible patients with sufficient tumor tissue, we performed tumor/normal genome-wide exome sequencing to identify somatic alterations. Eleven patients had available tumor RNA that was successfully analyzed by RNA-sequencing. Sequencing metrics and summary statistics for each of the eligible patients are shown in Table 1.

**Table 1:** NGS sequencing metrics.

Patients	Tumor Exome Aligned Reads (millions)	Tumor Exome Average Target Coverage	Tumor Exome >100X Target Coverage	Normal Exome Aligned Reads (millions)	Normal Exome Average Target Coverage	Tumor RNA Aligned Reads (millions)	Tumor RNA % mRNA Bases^a^
**SCLC001**	360	310X	92%	166	158X	325	87%
**SCLC002**	235	222X	82%	116	118X	170	87%
**SCLC003**	361	337X	92%	160	156X	240	91%
**SCLC004**	406	386X	93%	164	161X	282	91%
**SCLC006**	436	423X	94%	199	200X	319	90%
**SCLC007**	481	505X	94%	238	267X	218	88%
**SCLC008**	458	460X	93%	216	228X	202	89%
**SCLC009**	557	560X	96%	243	244X	172	90%
**SCLC010**	301	326X	90%	155	173X	377	88%
**SCLC011**	526	540X	95%	187	210X	178	91%
**SCLC012**	514	534X	94%	247	270X	N/A	N/A
**SCLC013**	449	441X	95%	202	222X	250	92%

N/A, not available.

^a^Percent of aligned bases that map to coding regions and UTRs of mRNA transcripts.

Tumor content averaged >80% and ranged from 20–100% based on pathology review. The minimum tumor content for successful NGS was 20%. The median turnaround time from sample collection to report was 15 days (range 11–17). The median turnaround time from sample collection to a genomics-based treatment recommendation by the molecular tumor board was 27 days (range 21–39). The median time to initiation of NGS guided treatment for the three patients that received NGS guided therapy after the molecular tumor board recommendation was 32 days (range 30–59). For the six patients that did not receive NGS guided therapy, the median time to clinical deterioration from the molecular tumor board recommendation was 10 days (range 0–45) (Table 2).

**Table 2:** NGS turnaround time metrics.

Patient Number	Tumor content by Pathology	Days from samples received to date of report	Days from samples received to molecular tumor board recommendation	Patient received NGS guided treatment?	Days from disease progression (PD) on previous therapy to start on NGS guided treatment or clinical deterioration
**SCLC0001**	20%	16	21	Yes	59
**SCLC0002**	99%	11	24	No	8
**SCLC0003**	75%	17	27	No	11
**SCLC0004**	50%	15	25	Yes	Prior to NGS result becoming available
**SCLC0006**	99%	15	27	No	0
**SCLC0007**	95%	17	24	Yes	30
**SCLC0008**	80%	16	28	Yes	Prior to NGS result becoming available
**SCLC0009**	80%	14	26	No	45
**SCLC0010**	100%	14	34	Yes	32
**SCLC0011**	98%	14	24	No	0
**SCLC0012**	100%	17	39	No	12
**SCLC0013**	98%	15	32	Yes	Prior to NGS result becoming available

**Fig 2:**
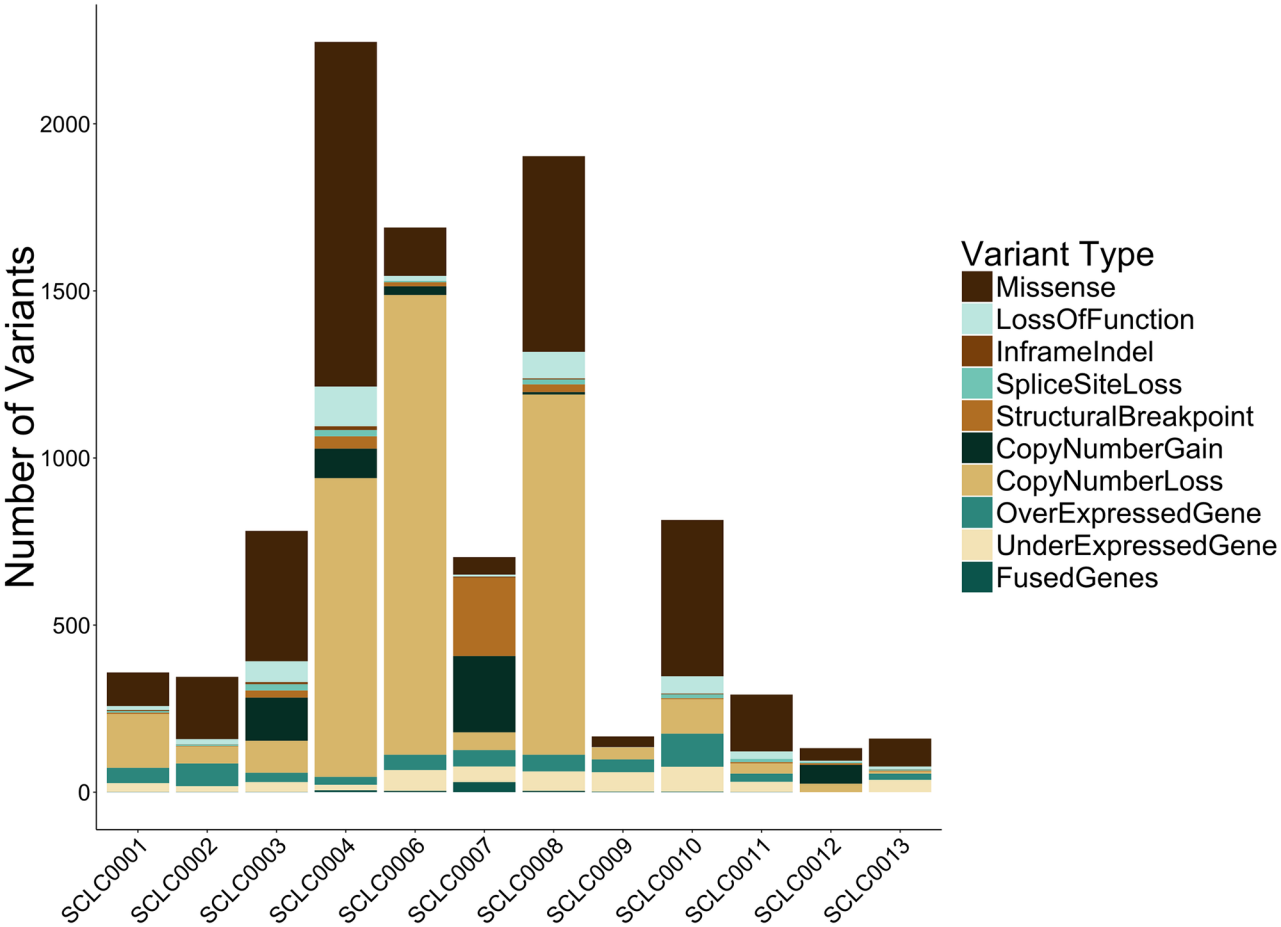
Genomic alteration overview. Summary of the number and types of somatic variants detected by tumor/normal genome-wide exome and tumor RNA-sequencing for each SCLC patient (n = 12).

Average Exome coverage was 420X (tumor), 201X (germline), with an average of 248 million aligned RNA reads generated for tumors. The number and type of genomic aberrations for each patient are depicted in Fig 2.

**Fig 3:**
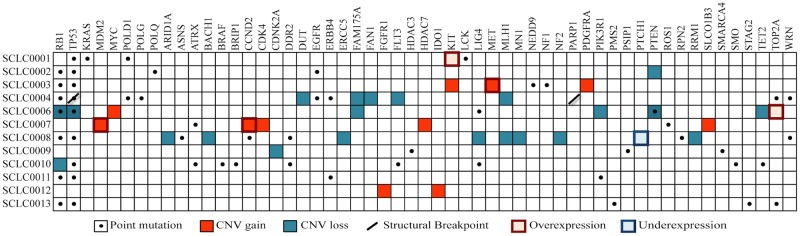
Summary of genomic alterations. Frequency and spectrum of selected alterations detected in advanced small cell lung carcinoma cases (n = 12). Genes previously reported to be recurrently altered in SCLC are presented, along with alterations deemed potentially therapeutically actionable.

Table 3 lists the clinical characteristics for the 12 eligible patients who consented for this study, and Fig 3 summarizes the molecular findings. As expected, TP53 and RB1 were recurrently altered in this cohort, with mutations and/or copy number loss of these genes co-occurring in two-thirds (8 out of 12) of the tumors (Fig 3). Potentially actionable alterations were observed in all patients, though few genes were recurrently altered in more than one patient. All patients had at least two clinically actionable targets identified (associated with a commercially-available, FDA-approved drug by predefined rules), with a median of three targets (range 2–11). S1 Table contains additional information on these potentially actionable genomic alterations.

**Table 3:** Clinical characteristics for the eligible patients.

Patient	Age at entry	Gender	Ethnicity	Cig hx	KPS at entry	Prior treatment	Platinum sensitive, resistant, or refractory	Prior brain mets?	Biopsy site
**SCLC0001**	74	F	Caucasian	Yes	90%	Carbo/etop	Resistant	No	Liver mass
**SCLC0002**	56	F	Caucasian	Yes	90%	Cis/etop	Sensitive	No	Liver mass
**SCLC0003**	68	M	Caucasian	Yes	80%	Irino	Refractory	No	Right lung mass
**SCLC0004**	57	M	Caucasian	Yes	90%	Carbo/etop	Resistant	No	Adrenal mass
**SCLC0006**	51	F	Caucasian	Yes	80%	Cis/etop	Refractory	No	Cervical lymph node
**SCLC0007**	43	F	Native American	No	90%	Cis/etop/XRT/topo	Sensitive	No	Right lower lung mass
**SCLC0008**	60	M	Caucasian	Yes	90%	Carbo/etop	Resistant	No	Left supra lymph node
**SCLC0009**	57	F	Caucasian	No	90%	Carbo/etop, /topo	Refractory	Yes	Liver mass
**SCLC0010**	66	F	Caucasian/Hispanic	Yes	90%	Carbo/etop, /topo, pacli	Resistant	No	Left lower lung mass
**SCLC0011**	54	F	Caucasian	Yes	70%	Cis/etop	Refractory	Yes	Left cervical jugular lymph node
**SCLC0012**	55	F	Caucasian	No	80%	Cis/etop/XRT/topo	Sensitive	No	Right preauricular mass
**SCLC0013**	54	F	Caucasian	Yes	80%	Cis/etop/XRT	Refractory	Yes	Liver mass

M, male; F, female; Cig hx, smoking history; KPS, Karnofsky Performance Status; carbo, carboplatin; etop, etoposide; cis, cisplatin; irino, irinotecan; XRT, radiation; topo, topotecan; pacli, paclitaxel; mets, metastases; supra, supraclavicular.

**Fig 4:**
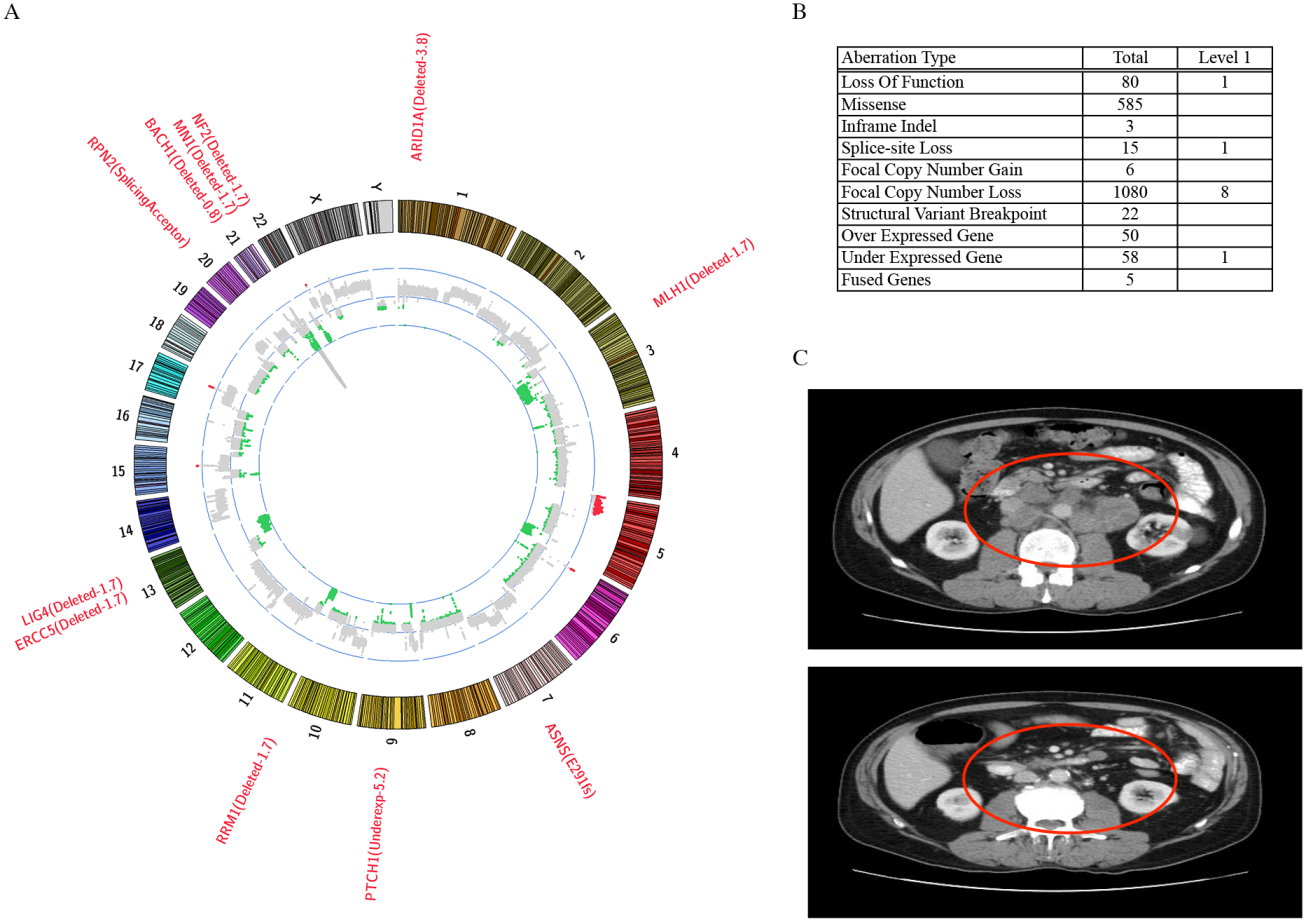
Representative example. (A) Circos plot for SCLC0008, with potentially actionable alterations listed in red font. (B) Summary of the type and number of genomic aberrations detected for SCLC0008. Level 1 corresponds to alterations viewed as potentially clinically actionable. (C) A representative baseline radiographic image (top image) and a response evaluation scan at 11 months (bottom image) for SCLC0008, with the red circle surrounding tumor burden in the retroperitoneum and left adrenal gland. The overall tumor burden decreased from 10.8 cm to 2.3 cm, with CEA tumor marker decreasing from 94.8 to 7.8.

Six (50%) patients received treatment identified by NGS (Table 4). For three of these patients, their treatment linked to NGS was already initiated prior to the NGS report becoming available. Of these, two had partial responses by RECIST 1.1 >10 months on a clinical trial involving PD-1 inhibitor + irinotecan (indicated by *MLH1* molecular alterations) (Representative example in Fig 4). Three had disease progression (PD) as best overall response: one on PD-1 inhibitor (indicated by a *PMS2* mutation), one on dasatinib (indicated by KIT overexpression), and one on temozolomide (indicated by *LIG4* copy number loss). The remaining six evaluable patients had clinical deterioration before NGS recommended therapy could be initiated.

**Table 4:** Summary of treatment received and response.

Patient	Recommended therapy received	Best response
**SCLC0001**	Dasatinib	PD
**SCLC0002**	None	na
**SCLC0003**	None	na
**SCLC0004**	Irinotecan + PD1^a^	PR
**SCLC0006**	None	na
**SCLC0007**	Irinotecan	SD
**SCLC0008**	Irinotecan + PD1^a^	PR
**SCLC0009**	None	na
**SCLC0010**	Temozolomide	PD
**SCLC0011**	None	na
**SCLC0012**	None	na
**SCLC0013**	Irinotecan + PD1^a^	PD

PD, disease progression; SD, stable disease; PR, partial response; na, not applicable.

^a^Treatment started prior to genomic results being available.

Three of the twelve patients were treated with a PD-1 inhibitor in combination with irinotecan as part of a separate, ongoing clinical trial (NCT02331251). Two of the three demonstrated partial responses by RECIST 1.1 criteria, while the other patient had PD as a best response. In the two patients with partial responses (SCLC0004, SCLC0008), exome-sequencing identified 1031 and 585 somatic missense mutations (Fig 2) as well as molecular alterations in *MLH1* in both tumors (Fig 3). These 2 patients had the highest mutational burdens across the cohort (average mutation count: 273). The patient that progressed on PD-1 + irinotecan (SCLC0013) harbored 83 missense mutations, and was linked to PD-1 by an alteration in *PMS2*.

## Discussion

This pilot study aimed to demonstrate feasibility of the employment of NGS-guided treatment in advanced SCLC, an effort to bring us closer to precision medicine for SCLC. Results generated from this work use a novel approach to identify and characterize new contexts of vulnerability for both current therapies and agents in development, thus enabling accelerated clinical implementation. Completion of the work proposed here will generate additional support for future precision medicine studies in SCLC to the benefit of more patients with this devastating cancer. We will develop and validate this approach to include a flexible cadre of cutting-edge genomic technologies that will also serve to inform precision medicine for other cancer types, leveraging the current study design as a template.

Conducting novel pilot studies using fresh tumor tissue from advanced cancer patients is not a new endeavor for our group. The SCRI-CA-001 “Bisgrove” Trial was a prospective, open-labeled, nine-center study designed to determine whether molecular profiling tumors from patients with advanced, previously treated cancer could provide any benefit to patients [20]. To be eligible, patients must have had 2–4 prior chemotherapies or hormonal or biologic regimens for their advanced disease, measurable or evaluable refractory disease, and clear documentation of the time between initiation of treatment and documented progression on the last treatment prior to study entry. Patients with all histologic types of malignancy were allowed on study. The patients were treated based on the results generated in the molecular profiling. The primary objective was to determine the growth modulation index (GMI) of treatment regimens selected by profiling of tumor biopsies. Molecular profiling was deemed of clinical benefit for the individual patient who had a GMI ratio >1.3. This was the first published study utilizing molecular profiling to find potential target and select treatments accordingly. Eighty-six patients were profiled with a molecular target identified in 84 (98%). Sixty-six patients were treated according to the profiling results of which 18/66 (27%) had a PFS ratio advantage ≥ 1.3 [20]. There were also one complete and five partial responses, as well as, 14 patients without progression at 4 months. In most patients, successful tumor profiling supported the indication of a new treatment not contemplated initially by the physician.

The results from the NFCR trial demonstrate that we have the ability and experience to conduct not only WGS in advanced cancer patients, but also whole-transcriptome sequencing (WTS) with integrated DNA and RNA analyses [16]. This advancement demonstrates improvement in our next generation sequencing pipeline, which now also involves tumor DNA and RNA isolation within our in-house CLIA-certified diagnostic laboratory, improving the turn-around time from analyte extraction and quality control to interpreted sequence data. As the reagent chemistry for sequencing has improved, we can now perform GWS with sufficient coverage even when the tumor comprises only 20% of the total sample volume. With these collective resources, we are poised to apply our state-of-the-art technologies to patients with advanced SCLC, as well as other advanced tumor types.

A takeaway lesson from both the Bisgrove and NFCR studies was the importance of turnaround time of results to be able to act upon the NGS guided recommendation for patients with advanced/metastatic and recently progressed disease. To that end, all patients in this trial were offered next line therapy while whole exome and transcriptome sequencing was being performed to allow for sufficient time to get to the recommendations when they would be needed. Somewhat surprising is that despite this strategy, half of the patients still had clinical deterioration making them ineligible for NGS guided therapy. Getting a NGS report turnaround sooner would not have made a difference clinically for these six patients. One possible solution for future studies involving SCLC patients, would be earlier access to NGS guided therapy. With continuing improvements in pricing, employing NGS during first-line therapy coupled with a proactive approach to securing coverage/approval for NGS guided therapy or the identification of an appropriate clinical trial based on the NGS findings, may allow for a higher percentage of patients getting NGS guided therapy. Only with a sufficient fraction of the study population receiving NGS guided therapy, can the potential value of its application be measured.

The success of immune checkpoint inhibitors has led to use of these therapeutics across a wide range of tumor types. Blockade of CTLA4 and/or PD-1/PD-L1 have shown clinical successes in melanoma, lung cancer, and other tumor types [21, 22]. In SCLC, a number of completed or ongoing clinical trials (reviewed in [5, 13]) have demonstrated clinical efficacy with these immune-based therapies alone or in combination with chemotherapy. A phase I/II study (CheckMate 032) employing nivolumab with or without ipilimumab in recurrent SCLC demonstrated an overall response rate of 10% or 19–23%, respectively [15]. There was no correlation between PD-L1 expression and response. In another phase 1b trial, PD-L1-positive patients with extensive stage SCLC were treated with pembrolizumab, and displayed an overall response rate of 35%, although again with no significant relationship between level of PD-L1 expression and response [14]. Thus, while success of PD-1 inhibitors in SCLC has been demonstrated, markers of response/resistance are lacking. In this study, two of three patients had partial responses on PD-1 inhibition in combination with irinotecan. It should be emphasized that their treatment was already initiated prior to the NGS report becoming available. The two responders had molecular alterations in *MLH1* and the highest frequencies of missense mutations. The patient with PD had an alteration in *PMS2*, and a low mutational burden. Previous work has shown correlations between immune therapy response and mutational burden, including a molecular smoking signature [23, 24]. Molecular alterations in *MLH1* also correlated with response to PD-1 inhibitors or PD-L1 protein staining [25, 26]. Our data suggest that a combination of molecular alteration and mutational burden may better predict response to PD-1 inhibition, molecular outcomes afforded by deeper sequencing techniques. Future work will explore these combinational biomarkers to determine immune therapy response.

In conclusion, this pilot trial successfully recruited the goal of 12 patients with SCLC that progressed on frontline chemotherapeutic options. Next-generation sequencing revealed potential therapeutic targets in all 12 patients. SCLC after first-line therapy tends to have more rapid progression and deterioration making NGS application for systemic therapy challenging. Two patients demonstrated partial responses on a combinational regimen of an immune checkpoint inhibitor + irinotecan, a regimen post-hoc revealed by NGS and suggested by mismatch repair gene alterations. Earlier access to NGS guided therapy, along with a better understanding of those SCLC patients likely to respond to immune-based therapies should help to extend survival in these cases with poor outcomes.

## Supporting information

S1 Table Summary of potentially actionable genomic alterations. Detailed information on potentially therapeutically actionable alterations identified in 12 SCLC tumors.
(XLSX)

S1 File Protocol. The protocol for this pilot study.
(PDF)

S2 File TREND checklist. The completed TREND checklist submitted for this manuscript.
(PDF)
