# Supplementary material for: A prospective pilot study of genome-wide exome and transcriptome profiling in patients with small cell lung cancer progressing after first-line therapy
Source: PLoS One. 2017 Jun 6;12(6):e0179170. doi: 10.1371/journal.pone.0179170 (PMC5460863; doi:10.1371/journal.pone.0179170)
Supplement: S1 File — The protocol for this pilot study. (PDF) [file pone.0179170.s002.pdf]

# TRANSLATIONAL GENOMICS RESEARCH INSTITUTE

## CLINICAL PROTOCOL

**Protocol Title:** Next-generation sequencing of Small Cell Lung Cancer to Identify Actionable Targets for Treatment

**Short Title:** Targets for Treatment Using NGS in Small Cell Lung Cancer

**Investigators:** Glen Weiss, M.D.
Director of Clinical Research,
Western Regional Medical Center, Inc.
14200 W. Celebrate Life Way
Goodyear, AZ 85338

Sub Investigator:
John Carpten, Ph.D.
TGen
445 N 5th Street, Suite 500
Phoenix, AZ 85004
602-343-8819

**TGen Protocol Number:** Gweiss13-007

**Sponsor:** Translational Genomics Research Institute (Tgen)

**Protocol Version Date:** 26 MAY 2014

**Previous Version (Date):** 29 APR 2013

05 DEC 2013

**Confidentiality Statement**

This document is confidential and is to be distributed for review only to investigators, potential investigators, consultants, study staff, and the applicable independent ethics committees or institutional review boards. The contents of this document shall not be disclosed to others without written authorization from Dr. Glen Weiss unless it is necessary to obtain informed consent from potential study participants.

## PROTOCOL SYNOPSIS

**Name of Sponsor:** CTCA
**Protocol Title:** Next-generation sequencing of Small Cell Lung Cancer to Identify Actionable Targets for Treatment

**Study Objectives:**

***Aim 1 – Launch Pilot Study.*** In this aim, we seek to launch a pilot study and enroll 12 eligible patients with advanced small cell lung cancer (SCLC) and to obtain the necessary tumor biopsies to yield sufficient DNA and RNA for Genome-Wide Sequencing (GWS).

***Aim 2*** **– Treatment Selection.** Completion of this study aim will provide a new clinical paradigm in the treatment of SCLC such that each individual patient would be treated with a single-agent or combination therapy of commercially available agents that relates to particular target(s) that have been identified via GWS.

**Study Design:**

***Aim 1 – Pilot Study.*** *Case subjects*: Up to 12 patients will be enrolled who meet the eligibility criteria for advanced SCLC. To be eligible, patients must have received at least one line of prior chemotherapeutic or biological regimens for advanced disease and have measurable or evaluable disease. Eligible patients must undergo or have available a tumor biopsy within 8 weeks. If the patient is ineligible to receive systemic therapy when GWS results become available, the results will be discussed with patient (or next of kin) and the referring oncologist.

***Aim 2 – Treatment Selection.*** Participants for this arm of the study will be treated based on target(s) identified via GWS. Potential targets (see Appendix 1) will be based on recent reports of whole-genome sequencing (WGS) of lung cancer, which may include MYC aberration (bortezomib), EGFR mutation (erlotinib), and PTEN deletion (everolimus or temsirolimus).

**Number of Subjects:**
Up to a total of 12 subjects will be enrolled in the study.

**Duration of Study Participation:**
Participation in this study will begin at the time the subject is enrolled and will end after once treatment selection has been made based on the data from GWS results.

## LIST OF ABBREVIATIONS AND DEFINITIONS OF TERMS

| Abbreviation | Definition |
|---|---|
| CRF | Case Report Form |
| CTCA | Cancer Treatment Centers of America |
| GCP | Good Clinical Practice |
| GWS | Genome-Wide Sequencing |
| HIPAA | Health Insurance Portability and Accountability Act |
| IATA | International Air Transport Association |
| ICH | International Conference on Harmonization |
| IRB | Institutional Review Board |
| NSCLC | Non-small cell lung cancer |
| PHI | Protected Health Information |
| SCLC | Small cell lung cancer |
| TGen | Translational Genomics Research Institute |
| WGS | Whole-genome sequencing |
| WTS | Whole-transcriptome sequencing |

# 1 BACKGROUND AND RATIONALE

Lung cancer is the leading cause of cancer deaths in both women and men in the United States and throughout the world. The two main types of lung cancer are non-small cell lung cancer (NSCLC) and small cell lung cancer (SCLC). SCLC accounts for ~12% of all lung cancers, and more than 27,000 new cases of SCLC are diagnosed annually in the U.S. For SCLC patients who progress after first-line therapy, the standard FDA-approved treatment options are either topotecan or the combination of cyclophosphamide, doxorubicin, and vincristine. For second-line therapy, the overall response rate is 22-44%, with a median progression-free survival (PFS) and overall survival of 3.3 months - 4.5 months and 7.6 months - 9.2 months, respectively.

PFS rates are dismal in these patients and no significant progress in the treatment for advanced SCLC has been made for decades. In this protocol, therefore, we address this critical clinical need, concentrating on patients with SCLC and evaluating genome-wide sequencing (GWS) and integrated analysis to identify tumor-specific targets for chemotherapeutics that will directly translate into improved treatment outcomes and reduced morbidity and mortality for patients with this lung cancer. The work proposed herein is highly significant because these patients often have few, if any, treatment options, and some may not be eligible for investigational agent trials if beyond first-line therapy (Figure 1).

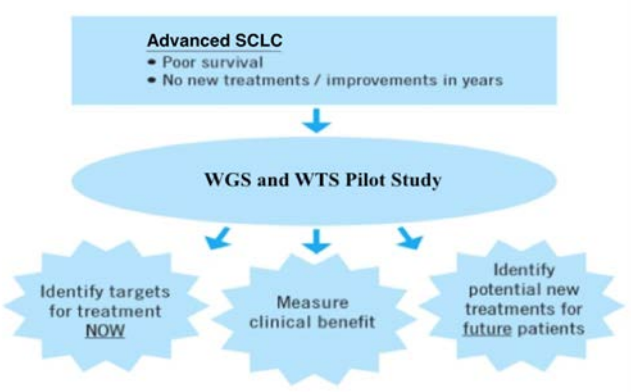


In this pilot study, we will specifically use novel methods such as GWS and integrated analysis for individual advanced SCLC patients in a prospective manner. For advanced SCLC cases that progress on standard therapies, identification of damaging, non-synonymous somatic mutations, and the biological processes they regulate, will be a major advance toward individualizing therapy and improving the outcome of these patients. **This pilot study seeks to have a transformative impact on understanding the molecular factors that lead to progression of advanced SCLC after at least one line of chemotherapy**.

The information to be garnered from this pilot study and its effort to bring us closer to precision medicine for SCLC. Results generated from this work use a novel approach to identify and characterize new contexts of vulnerability for both current therapies and agents in development, thus enabling accelerated clinical implementation. Completion of the work proposed here will generate additional support for future precision medicine studies in SCLC to the benefit of more patients with these devastating cancers. We will develop and validate this approach to include a flexible cadre of cutting-edge genomic technologies that will also serve to

inform precision medicine for other cancer types, leveraging the current study design as a template.

The focus of the current study is to improve treatment strategies for individuals with SCLC. We have recently completed a pilot study (NFCR trial) for patients with advanced rare cancers (NCT01443390) to evaluate the utility of identifying potential therapeutic targets in tumors from patients whose cancers had progressed on standard therapies. Our preliminary findings in advanced cancer using GWS indicate at least one actionable target with a conventional FDA-approved agent or drug in clinical development. In this study, we propose a similar approach to the investigation of SCLC. Through the use of state-of-the-art techniques, we aim to launch the pilot study, enroll eligible advanced-SCLC patients, and complete GWS analyses prospectively to determine somatic aberrations that could be targeted by an existing therapy. Completion of this study will aim to provide a new clinical paradigm in the treatment of SCLC such that each individual patient would be treated with a single-agent or combination therapy with commercially-available agents that relates to particular targets identified via GWS.

We know that somatic alterations (i.e., point mutations, small insertions and deletions, rearrangements, gains and losses) occur at the DNA level in cancer. These somatic events can drive tumorigenesis, metastatic progression, and/or drug resistance. More importantly, specific somatic alterations can be targeted by therapeutics. For the first time, technology now offers us the ability to survey the global somatic landscape of cancer: it is possible with GWS to sequence, analyze, and compare the matched tumor and normal genomes of an individual patient. In order to take full advantage of these advances, we intend to apply such capabilities in the clinic and translate them into the management of individual patients.

Conducting novel pilot studies using fresh tumor tissue from advanced cancer patients is not a new endeavor for our group. The SCRI-CA-001 “Bisgrove” Trial was designed to determine whether molecular profiling of tumors at an advanced, previously treated stage could provide any benefit to patients. The trial was a prospective, open-labeled, nine-center study for patients with advanced solid tumors. To be eligible, patients must have had 2-4 prior chemotherapies or hormonal or biologic regimens for their advanced disease, measurable or evaluable refractory disease, and clear documentation of the time between initiation of treatment and documented progression on the last treatment prior to study entry. Patients with all histologic types of malignancy were allowed on study. The patients were treated based on the results generated in the molecular profiling. The primary objective was to determine the growth modulation index (GMI) of treatment regimens selected by profiling of tumor biopsies. Molecular profiling was deemed of clinical benefit for the individual patient who had a GMI ratio >1.3. This was the first published study utilizing molecular profiling to find potential target and select treatments accordingly. Eighty-six patients were profiled with a molecular target identified in 84 (98%). Sixty-six patients were treated according to the profiling results of which 18/66 (27%) had a PFS ratio advantage $\geq$ 1.3. There were also 1 complete and 5 partial responses, as well as 14 patients without progression at 4 months. In most patients, successful tumor profiling supported the indication of a new treatment not contemplated initially by the physician.

The results from the NFCR Trial demonstrate that we have the ability and experience to conduct not only WGS in advanced cancer patients, but also whole-transcriptome sequencing (WTS) with integrated DNA and RNA analyses. This advancement demonstrates improvement in our next generation sequencing pipeline, which now also involves tumor DNA and RNA isolation within our in-house CLIA-certified diagnostic laboratory, improving the turn-around time from analyte extraction and quality control to interpreted sequence data. As the reagent chemistry for sequencing has improved, we can now perform GWS with sufficient coverage even when the tumor comprises only 30% of the total sample volume. With these collective resources, we are poised to apply our state-of-the-art technologies to patients with advanced SCLC.

# 2 STUDY OBJECTIVES

***Aim 1 – Launch Pilot Study.*** In this aim, we seek to launch a pilot study and enroll 12 eligible patients with advanced SCLC, and to obtain the necessary tumor biopsies to yield sufficient DNA and RNA for genome-wide interrogation.

***Aim 2 –* Treatment Selection.** Completion of this study aim will provide a new clinical paradigm in the treatment of SCLC such that each individual patient would be treated with a single-agent or combination therapy of commercially available agents that relates to particular target(s) that have been identified via GWS.

# 3 STUDY POPULATION

## 3.1 Source and Number of Subjects

Up to 12 patients will be enrolled who meet the eligibility criteria for advanced SCLC.

## 3.2 Subject Selection

This will be a single-center pilot study for patients with advanced SCLC. This protocol will be run and patients will be enrolled at Western Regional Medical Center (WRMC) at Cancer Treatment Centers of America (CTCA). Eligible patients will undergo tumor biopsy at WRMC, and subsequent systemic treatment will be delivered at WRMC. To qualify for participation, all subjects must meet all of their respective inclusion criteria and none of their respective exclusion criteria.

### 3.2.1 Inclusion and Exclusion Criteria

- **Inclusion Criteria:**
  - Patients must understand the rigors of the study and provide written informed consent and HIPAA authorization prior to initiation of any study procedures
  - Life expectancy > 3 months
  - Karnofsky Performance Status ≥ 70

- Diagnosis of histological or cytologically confirmed advanced, incurable SCLC, which has progressed on one or more prior chemotherapeutic, hormonal, or biological regimens for advanced SCLC
- Age ≥ 18 years
- Adequate organ and bone marrow function, defined as:
- Bone marrow: absolute neutrophil count (ANC) ≥ 1.5 x $10^9$/L; hemoglobin > 9 g/dL; platelets > 100 x $10^9$/L
- Renal: creatinine clearance ≥ 50 mL/min (calculated according to Cockroft and Gault) or creatinine ≤ 1.5 mg/dL
- Hepatic: bilirubin ≤ 1.5 x the upper limit of normal (ULN); aspartate transaminases (AST/SGOT) and alanine transaminases (ALT/SGPT) ≤ 2.5 x ULN (or ≤ 5 x ULN if due to underlying liver metastases); internation normalized value for prothrombin time (INR) ≤ 1.5 x ULN (except in the case of anticoagulation therapy), albumin ≥ 2.0
- Good medical candidate for and willing to undergo a biopsy or surgical procedure to obtain tissue, which may or may not be part of the patient's routine care for their malignancy.

Note: Patients in this trial can participate on a non-therapeutic clinical study prior to or during this trial.

- **Exclusion Criteria**
  - Symptomatic CNS metastasis. Patients with a history of CNS metastases, who have been treated, must be stable without symptoms for 4 weeks after completion of treatment, with image documentation required, and must be either off steroids or on a stable dose of steroids for ≥ 2 weeks prior to enrollment
  - Uncontrolled intercurrent illness including, but not limited to, ongoing or active serious infection, symptomatic congestive heart failure, unstable angina pectoris, unstable cardiac arrhythmias, psychiatric illness, or situations that would limit compliance with the study requirements or the ability to willingly give written informed consent
  - Known HIV, HBV, or HCV infection requiring antiviral therapy.
  - Pregnant or breastfeeding patients or any patient of childbearing potential not using adequate contraception.
  - Tumor inaccessible for biopsy

### 3.3 Subject Withdraw

Patients may withdraw from the study at any time. If a patient withdraws from the study, the data and samples already collected will not be removed from the study database, but no further data will be collected or added to the database. With signed informed consent, samples sent to TGen will be stored without identifiers for an indefinite period of time and will be used for future research.

## 4 CLINICAL STUDY PROCEDURES

### 4.1 *Specimen Collection*

Potential patients will be enrolled at WRMC when they present for care of their advanced SCLC. All patients must have signed and dated the informed consent and HIPAA Authorization prior to any study specific screening procedures being performed. Pre-enrollment screening test and evaluations will be used to determine the eligibility of the patient for study inclusion. All screening tests and procedures must be completed within 28 days prior to study registration and include the following:

1. Demography and Medical History
2. Physical exam including height and weight
3. Vital signs including: temperature, BP, pulse, respiration rate
4. PT, INR,
5. Review of radiological scans to assess accessible tumor for biopsy
6. If the patient is determined to be eligible, the patient will be enrolled in the study and assigned a patient study number. A tumor biopsy will be scheduled.

After informed consent is signed and potential participants are found to meet the entry criteria for this study, eligible patients will undergo a biopsy of an accessible lesion. Fresh frozen tumor specimens obtained by the biopsy will be collected in liquid nitrogen immediately upon collection. Tumor biopsy samples must be submitted with a pathology report.

WRMC will add the patient's study ID and Date of Birth to the all samples sent to the CLIA Laboratories. Both identifiers will be required in order for the CLIA laboratory to accept the samples.

If available, this study will collect FFPE block(s) of tumor tissue from prior surgery or biopsy. If an entire block is not available slices of the block totaling 100 microns will be collected form the tumor block.

Additionally, approximately 6mL of peripheral blood will be collected to isolate germline DNA. Blood will be collected in a purple top EDTA tube.

| **Tissue Required** | **Amount/Container** | **Intended Use** |
|---|---|---|
| Tumor tissue | Fresh frozen tissue<br><br>Paraffin embedded tumor block (30 slides) | GWS<br><br>Correlative studies |
| Whole blood | One 6 mL purple top EDTA tube | GWS |

Provided samples are adequately stored and processed within 90 days of consenting for this study, a new tumor biopsy will not be needed. The specific tissue collection, handling and shipping instructions will be provided by TGen. The blood tube will be stored cold **(not frozen)** until shipping with cold packs by overnight

courier to the CLIA certified Laboratory (Ashion).. Frozen tumor tissue will be shipped with dry ice to Ashion. Kits and shipping instructions will be provided.

**Schedule of Events:**

- Participants will be consented into the study after they are found to meet the study inclusion criteria.
- The clinical staff will schedule a tissue biopsy and a blood draw for the participant. Clinical staff will try to leverage a clinically indicated blood draw if possible. However, a research specific blood draw can be scheduled if needed.
- Approximately 6mL of blood will be collected in a purple top EDTA vacutainer tube.
- Blood will be sent to Ashion for DNA extraction.
- Tumor specimens will be shipped to Ashion for DNA and RNA extraction.
- Ashion will process the samples for DNA and RNA Sequencing in their CLIA certified laboratory. A portion of the nucleic acid will be stored in Ashion and used to confirm actionable targets.
- If previously acquired FFPE tumor blocks are available from prior surgery or biopsy, these will be obtained for possible correlation with sequence findings on the specimen biopsied for this study.
- Results will be submitted to the physician for possible inclusion in the treatment regimen.

## 4.2 Data Collection Forms

Data Collection Forms (DCF) will be used to collect clinically annotated data, such as medical history. The clinical research coordinator is responsible for maintaining accurate, complete and up-to-date records for each patient. All DCFs should be completed in a neat, legible manner to ensure accurate interpretation of the data.

### 4.3 Identification of Targets and Selection of Therapy

After sequencing analysis has been performed, a report will be provided to the treating oncologist. The PI and the treating oncologist may review the results to identify potential treatment (for example see Appendix 2). The treating oncologist may use this information at their discretion, and is not required to treat the patient based on the targets identified by the GWS analysis.

### 4.4 Follow-up

All enrolled patients will be contacted for cancer treatment history and survival status at 4, 8, 12, 18 and up to 24 months or until 12 months from last patient enrolled; whichever is shorter.

### 4.5 Specimen Handling

Appendixes 2, 3 and 4 outline proper specimen handling and shipping instructions for all specimens collected in this study.

## 5 LABORATORY PROCEDURES

### 5.1 Ashion Analytics

The CLIA-certified laboratory (Ashion Analytics, LLC) is a CLIA-certified laboratory that was developed by TGen. The sequencing and data analysis will be done by Ashion to allow for treatment decisions to be made from the data.

### 5.2 Whole Genome and Transcriptome Interrogation

Profiling will utilize Next Generation Sequencing technologies (NGS). We will employ a normal/tumor paired study design to capture 1) somatic mutations using whole genome sequencing, and 2) RNA-sequencing (RNA-seq) to generate gene and exon level expression profiles and to search for candidate oncogenic fusion transcripts. Integration of data will allow us to study allele specific expression (whole genome and RNA-seq). TGen has considerable expertise in the area of genomic interrogation of tumors for identifying somatic mutations related to targeted therapeutics, including in the area of breast cancer, as evidenced by our publication record (also see Figure 1). Our group was the first to use whole genome sequencing to identify therapeutically actionable events in patients with breast cancer (Craig et al., MCT, 2013).

For this project, we will leverage our Illumina NGS infrastructure. The Illumina HiSeq sequencing platforms enable scientific discovery with a full spectrum of applications, ranging from whole genome and targeted re-sequencing, to gene regulation analysis, single nucleotide polymorphisms (SNPs) discovery and structural variation analysis, cytogenic analysis, DNA-protein interaction analysis, small RNA discovery and analysis, linkage analysis, FFPE sample analysis, expression analysis, genotyping, and sequencing-based transcriptome or

methylation analysis. The Illumina NGS system utilizes two flow cells, each with eight sequencing ‘lanes’ where samples are loaded. It currently has the industry’s highest sequencing output and the fastest generation rate. This platform provides the investigator with previously unmatched cost savings and turnaround time for performing a wide variety of in-house experiments.

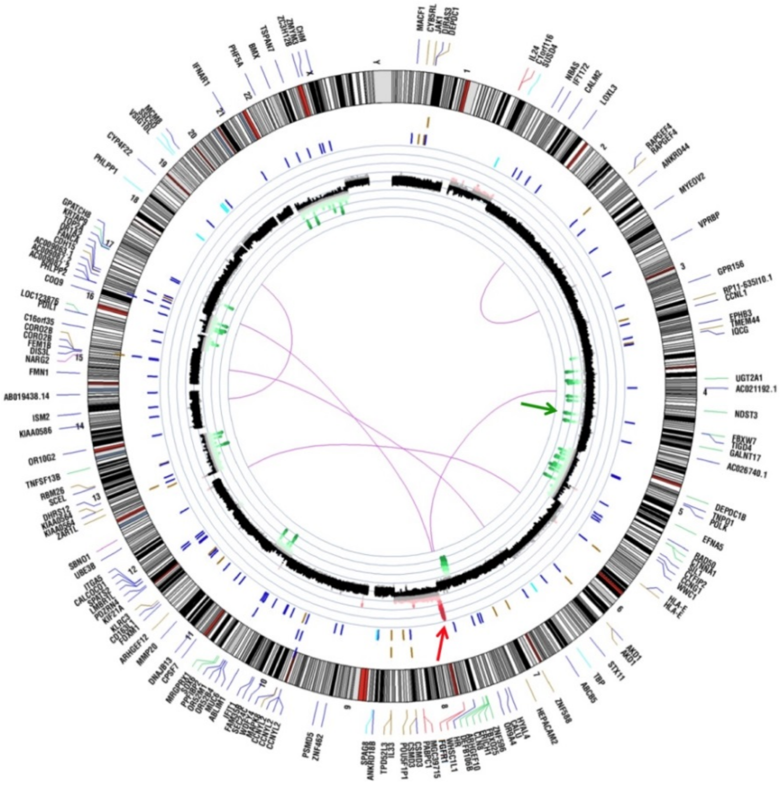

**Figure 1.** Circos plot showing somatic events in TNBC tumors including one with homozygous deletion of FBXW7 (green arrow) and amplification of FGFR1 (red arrow) (Craig et al., MCT 2013).

TGen has existing collaborations with Illumina with whom we have jointly published methods-development articles. Rather, our approach is more reliant on sequence throughput per lane where barcoded sequencing is critical, noting that the first Illumina description of barcoded sequencing was at TGen. Furthermore, we were early access users of the Illumina v3 reagents, which allow us to generate >25Gb of paired-end sequence per Illumina HiSeq 2000 lane or roughly >400Gb of uniquely mappabe sequence per run. Furthermore, TGen is among a small number of laboratories with the Illumina HiSeq2500, which provides upwards of 100Gb of sequencing within a 24-hour period using a 2-lane system (50Gb per lane). We will likely utilize this system for this project. We will be able to barcode and sequence whole exome normal/tumor pairs and RNA in a single HiSeq 2500 lane.

## 5.3 Data Analysis

Analysis of next generation sequencing data looms as one of the largest challenges in informatics today, and has been the largest focus of TGen’s high-performance informaticians over the past three years. Analysis and annotation involves error-free storing, transferring, backing-up, and processing of terabytes of data–validated

processes for analysis are critical. Our existing data processing and analysis pipeline is the result of managing internally generated data, and as one of the major participants in the International 1,000 Genomes Project (1KG). As part of the 1000 Genomes Initiative, TGen is responsible for mapping all of the 1000 Genomes project and developing novel software tools for analysis of NGS data (Craig PI, NHGRI: "Data Visualization and Analysis of 1000 Genomes Data", 1U01HG005210-01).

Our genome variant analysis pipeline is based on more than one in-house and publicly available algorithm. The intersection of multiple callers represents a so-called 'gold-list' that is used to reduce false positives and statistical artifacts specific to any one approach. The union of multiple approaches will have much higher false-positives, but will be used to reduce false negatives by visualization of events in key actionable genes. Visual inspection will then be used to promote variants to the final reports. Our RNA-seq pipeline consists of several publicly available tools and algorithms including Cufflinks and Tophat for mapping, read count generation, and junction detection.

## 5.4 Data Management and Processing Infrastructure

TGen's current infrastructure is equipped to handle complete analysis of up to 1 Terabase of sequence data per month. Our pipeline contains: (1) Massive storage infrastructures featuring expandable Isilon technology; (2) Massive cluster computing capabilities (30.1 Teraflops) with one of the 2 largest infrastructures dedicated to biomedical computing; (3) High memory computing with a 576 Gb shared memory machine; and (4) and next-generation file transfer with Aspera, with speeds reaching 100Mb to most T1-capable institutes.

## 5.5 Deliverables mutations and differential expression for biomarker discovery

All files will undergo a series of quality control checks to insure proper completion of analysis. Figure 1 provides an example of the types of data that can be drawn from somatic tumor sequencing by TGen scientists. For each normal/tumor pair we will compile a list of somatic alterations including: 1) Somatic coding point mutations and frameshift mutations; and 2) Germline mutations or SNPs involved in high-risk cancer predisposition or drug metabolism. We will also include genes that become evident only from integrated analysis of exome and transcriptome analysis, such as an obviously inactivating frameshift or nonsense mutation in the retained allele. Lastly, genetic alterations known to be relevant to pharmacological treatment pathways will be annotated.

## 5.6 Knowledge Mining Approach.

The output of the whole cancer genome profiling for each of the patients on the clinical trial will produce complex data, which will be processed and analyzed by the Ashion/TGen bioinformatics team. Several types of events are possible. Knowledge mining will involve a combination of expert and computationally assisted knowledge mining and interpretation to extract meaningful and clinically useful insights that can

support the physician to select a therapy that is tailored to the patient's specific genomic context. Based on the TGen experience of applying this process across ~30 cancer patients, it is safe to say that every tumor is different and the best target-to-drug concept is highly patient specific. We can expect though that the type of alterations within the tumor will fall into several possibilities. First, we may identify mutations that are directly targetable by existing pharmaceuticals. Examples include the *BRAF* V600E mutation, which is a biomarker for positive vemurafenib response in melanoma, however it may confer resistance to cetuximab in metastatic colorectal cancer with EGFR amplification. Second, we may identify differentially expressed genes such as HER2 that are directly targeted by existing pharmaceutical agents (likely for specific indications). Third, we may identify differentially expressed genes and mutations speaking to a broader 'concept' such as inactivating PTEN events, which lead to activation of the PI3 kinase pathway for which inhibitors exist. Fourth, we may identify mutations or expression changes that are indicative of poor outcome for which no direct target exists (such as *MYC* alterations). Finally, we will often encounter cooperating events that lead to activation of multiple pathways, which has been previously shown in advanced cancers. This can lead to mechanisms of drug resistance for single agents, or provide an opportunity to develop rationale combination therapies. All of these will be available as a compilation of targetable and non-targetable molecular information. This information will be summarized and using our tumor board concept we will prioritize potential markers for actionable treatment discussion..

## 5.7 Correlative Studies

FFPE specimens may be used when necessary for other correlative studies of identified targets. Studies that may be required include:

- Clonal Profiling of Genomes: Identify the clonal cell lineages in tumor tissue by profiling the genomes of flow-sorted samples using 1000k aCGH.
- Gene Expression and targeted tumor sequencing: gene expression profiles will be generated and relation to therapy will be explored.
- Knowledge mining: Knowledge mine and identify highly selected molecular concepts that are associated with outcome to therapy

# 6 RISKS AND BENEFITS TO PARTICIPANTS

## 6.1 Potential Benefits

There may be a benefit to study participation, if a clinically relevant mutation is identified in the molecular analysis. Identification of novel mutations may not confer any immediate benefit to subjects, as the clinical relevance of the mutation will not be confirmed. However, there is the potential for identification of mutations that may provide clues to the participant's condition that may alter their healthcare management and treatment.

There may be future benefits to society in general, as this research will increase the general knowledge regarding cancers and cancer metastasis. The integration of

genomics research with traditional clinical information adds another dimension of information descriptive of an individual participant's biology.

### 6.2 Potential Risks

There is a potential for slight physical risk involved in participation in this study. Patients who are willing to donate a blood sample may undergo a study-specific blood draw, which is associated with a slight risk of bleeding, bruising and infection. The collection of tumor tissue may be done as part of clinical care. However the patient may need to have a biopsy performed specifically for this project. Only patients who are deemed by the clinical investigator as meeting all of the inclusion criteria can participate in this project and be scheduled for a tumor biopsy. All study-specific procedures will be performed by a qualified staff member at the collection site.

As in all research studies, it is possible that participants may misunderstand the purpose of the study, or may feel anxious or become anxious about the thought of participating in a research study in general. Genomic studies that generate information about subjects' personal health and medical condition can provoke anxiety and confusion.

### 6.3 Incidental Findings

During the course of data analysis, investigators may discover genetic information about the study participant that is not related to the current study (an incidental finding). Both clinical and laboratory investigators will consult with one another to discuss the appropriateness of disclosing such information. They will consult with additional colleagues if needed (e.g., a genetic counselor). When deciding what to disclose, the team will take into account the health implications of the finding, if the finding is clinically actionable, or provides a potential benefits that outweighs the risks of knowing. Patients will be referred for genetic counseling if germline incidental findings are returned. The costs of the counseling will not be covered by the study.

### 6.4 Confidentiality

Information about study patients will be kept confidential and managed according to the requirements of the Health Insurance Portability and Accountability Act (HIPAA). WRMC will maintain a master enrollment log containing patient names and their assigned study-specific numbers. WRMC study staff will also maintain all signed informed consent documents along with participant data collection forms. All of these documents will be stored in locked cabinet(s) at the WRMC. Only Dr. Weiss or the study coordinator(s) will have routine access to the master enrollment log and patient identifiable information. The informed consent form discloses the limits of confidentiality and the fact that subjects may be requested to disclose such information by a third party. This is a risk of participation in the study and the subjects will be informed of this risk.

The information placed on the DCFs will be de-identified and labeled with the subject’s study-specific number. Copies of the completed DCFs and de-identified pathology report will be sent along with the patient’s sample to Ashion for storage and processing. TGen research laboratory staff will not see any identifying patient information that could be linked to the sample. TGen research staff will receive only de-identified and coded information. All consent forms will be maintained by the study coordinator at WRMC and will not be shared with TGen. Ashion (the CLIA-certified Laboratory) will receive the patient's date of birth and the date the sample was collected, as required for sample submission on the Ashion requisition form. This information will be stored in the Ashion LIMS database and TGen researchers will not have access to this information.

Data from the study may be published in scientific journals. Publications will not include any participant identifiers.

# 7 DATA SHARING

## 7.1 Data Sharing

De-identified data may be published to a central data repository that allows qualified research scientists to access and look at the data (such as dbGap).

## 7.2 Dissemination and Publication of Results

No publications or disclosure of study results will be permitted except under the terms and conditions of a separate written agreement between CTCA and TGen. All investigators must have the opportunity to review and approve all proposed presentations or publications of the data from this study.

# 8 STUDY ADMINISTRATION

## 8.1 Data Monitoring

The Study Investigators will review any toxicities, unacceptable delays (e.g. tumor sequence taking more than 3 weeks to complete), patient concerns or technical failures if they occur and/or are reported. If the data reveals a change in the risk/benefit ratio, the PI, will notify the IRB. The PI will then review the data with the sub-investigators and forward any changes or protocol amendments to the IRB. All serious adverse events will be immediately reported to the IRB as outlined in the full protocol. All study participant information will be kept in a confidential manner by the assigning of a random number to each study participant. All data will be kept confidential as per HIPPA guidelines and policies. Any breach of confidentiality is a serious matter and conflicts with institutional policies and will be reported to the IRB. A cumulative summary of all adverse events occurring on this study and a report of the data safety and monitoring plan will be submitted to the IRB with the annual renewal report.

## 8.2 *Protocol Compliance and Revisions*

All revisions to the protocol will be sent to the IRB for approval. The Investigator should not implement any deviation or change to the protocol without prior review and documented approval/favorable opinion from the IRB of an Amendment, except where necessary to eliminate an immediate hazard(s) to study patients.

### 8.3 *Informed Consent*

The Investigator must ensure that patients or their legally authorized representatives are clearly and fully informed about the purpose, potential risks and other critical issues regarding clinical trials in which they volunteer to participate. Preparation of the consent form is the responsibility of the Investigator and must include all elements required by CFR 21 Part 50.25 and the local IRB.

### 8.4 *Records and Reports*

An Investigator is required to prepare and maintain adequate and accurate case histories designed to record all clinically significant observations and other data pertinent to the investigation on each individual treated with the investigational product or entered as a control in the investigation.

### 8.5 *Institutional Review Board (IRB)*

Before study initiation, the Investigator must have written and dated approval/favorable opinion from the IRB for the protocol, consent form, patient recruitment materials/process (e.g., advertisements) and any other written information to be provided to patients.

The Investigator should provide the IRB with reports, updates, and other information (e.g., Safety Updates, Amendments, and Administrative Letters) according to regulatory requirements and Institution procedures.

### 8.6 *Records Retention*

The Investigator must retain case report forms and source documents for the maximum period required by applicable regulations and guidelines, or Institution procedures.

If the Investigator withdraws from the study (e.g. relocation, retirement), the records shall be transferred to a mutually agreed upon designee (e.g., another Investigator, IRB).

### 8.7 *Study Discontinuation*

CTCA and TGen reserve the right to discontinue the study at any time.

## 9 CITATIONS

# 10 APPENDICES

Appendix 1: Commercially Available Agents

Appendix 2: Blood Collection and Processing

Appendix 3: Frozen Tissue Collection and Processing

## Appendix 1: Commercially Available Agents

**Table 1. FDA-approved Agents**

| Drug Name | Mechanism of Action | Approved Indication | Route |
|---|---|---|---|
| Pomalidomide | Immunomodulatory anti-neoplastic agent | Celgene; For the treatment of relapsed and refractory multiple myeloma, Approved February 2013 | IV |
| Regorafenib | Tyrosine kinase inhibitor (VEGFR2 and TIE2 TK) | Bayer Healthcare Pharmaceuticals; For the treatment of gastrointestinal stromal tumor, Approved February 2013 | Oral |
| Everolimus | mTOR inhibitor | Novartis Pharmaceuticals Corporation; For the treatment of renal angiomyolipoma associated with tuberous sclerosis complex, Approved April 2012 | Oral |
| Bosutinib | Tyrosine kinase inhibitor | Pfizer; For the treatment of Ph+ chronic myelogenous leukemia, Approved September 2012 | Oral |
| Cabozantinib | Pan Tyrosine kinase inhibitor | Exelixis; For the treatment of metastatic medullary thyroid cancer, Approved November 2012 | Oral |
| Vismodegib | Hedgehog pathway signaling inhibitor | Genentech; For the treatment of basal cell carcinoma, Approved January 2012 | Oral |
| Axitinib | VEGFR inhibitor | Pfizer; For the treatment of advanced renal cell carcinoma, Approved January 2012 | Oral |
| Carfilzomib | Proteosome inhibitor | Onyx Pharmaceuticals; For the treatment of multiple myeloma, Approved July 2012 | IV |
| Pazopanib | VEGFR inhibitor | GlaxoSmithKline; For the treatment of soft tissue sarcoma, Approved April 2012 | Oral |
| Enzalutamide | Androgen receptor inhibitor | Medivation; For the treatment of metastatic castration-resistant prostate cancer, Approved August 2012 | Oral |

| | | | |
|---|---|---|---|
| Brentuximab vedotin | Anti-CD30 antibody conjugate of anti-neoplastic agent (monomethyl auristatin E) | Seattle Genetics; For the treatment of Hodgkin lymphoma and anaplastic large cell lymphoma, Approved August 2011 | IV |
| Vandetanib | Tyrosine kinase inhibitor (VEGFR and EGFR) | AstraZeneca; For the treatment of thyroid cancer, Approved April 2011 | Oral |
| Ipilimumab | CTLA-4 blockade (immunomodulatory) | Bristol-Myers Squibb; For the treatment of metastatic melanoma, Approved March 2011 | IV |
| Vemurafenib | Inhibitor of the activated BRAFV600E gene product | Roche; For the treatment of BRAF + melanoma, Approved August of 2011 | Oral |
| Abiraterone acetate | Inhibitor of CYP17 (cytochrome p450 complex) | Centocor Ortho Biotech; For the treatment of prostate cancer, Approved May 2011 | Oral |
| Bendamustine Hydrochloride | Alkylating agent | Cephalon; For the treatment of Chronic lymphocytic leukemia and B-cell non-Hodgkin's lymphoma, Approved October 2008 | IV |
| Cabazitaxel | Pan Tyrosine kinase inhibitor | Sanofi Aventis; For the treatment of prostate cancer, Approved June 2010 | IV |
| Ofatumumab | Anti-CD20 monoclonal antibody | GlaxoSmithKline; For the treatment of chronic lymphocytic leukemia, Approved October 2009 | IV |
| Bevacizumab | Anti-VEGF monoclonal antibody | Genentech; For the treatment of renal cell carcinoma, Approved July 2009 | IV |
| Pralatrexate | Anti-folate | Allos Therapeutics; For the treatment of peripheral T-cell lymphoma, Approved September 2009 | IV |
| Romidepsin | Histone deacetylase inhibitor | Gloucester Pharmaceuticals; For the treatment of cutaneous T-cell lymphoma, Approved November 2009 | IV |

| | | | |
|---|---|---|---|
| Topotecan | Topoisomerase I inhibitor | GlaxoSmithKline; For the treatment of small cell lung cancer, Approved October 2007 | Oral |
| Ixabepilone | Tubulin and microtubulin stabilization | Bristol-Myers Squibb; For the treatment of breast cancer, Approved October 2007 | IV |
| Nilotinib | Bcr-Abl kinase, c-kit, and platelet-derived growth factor | Novartis; For the treatment of chronic myelogenous leukemia, Approved October 2007 | Oral |
| Temsirolimus | mTOR inhibitor | Wyeth; For the treatment of renal cell carcinoma, Approved May 2007 | IV |
| Alemtuzumab | Anti-CD52 monoclonal antibody | Berlex Laboratories; Injectable treatment of B-cell chronic lymphocytic leukemia, Approved May 2001 | IV |
| Arsenic Trioxide | DNA fragmentation and degradation of the fusion protein PML-RAR alpha | Cell Therapeutics; For the induction of remission and consolidation in patients with acute promyelocytic leukemia (APL), Approved September 2000 | IV |
| Doxorubicin HCl Liposome | DNA intercalating agent | Alza; Treatment for ovarian cancer that is refractory to other first-line therapies, Approved June 1999 | IV |
| Temozolomide | Alkylating agent | Schering Corporation; Treatment treatment of adult patients with refractory anaplastic astrocytoma, Approved August 1999 | Oral |

# Appendix 2: Blood Collection and Processing

Blood samples will be drawn from participants that have been through the informed consent process for participation in the research study. Blood samples will be obtained by personnel qualified to draw blood from participants at the collection site. The purpose of this document is to outline standardized procedures for Collection Sites to follow for blood collection.

Clinical sites will be provided with SOPs detailing the proper packaging and shipping vessels to be employed for each sample type to protect them from loss, damage, and temperature variations during shipment. These protocols include the use of insulated packing material, refrigerated gel packs, frozen gel packs, dry ice pellets, and liquid nitrogen as appropriate.

## I. SAFETY

Always use universal precautions when dealing with any blood samples. Dispose of all blood collection equipment in the appropriate receptacles at the collection site.

## II. MATERIALS & EQUIPMENT

1. Tourniquet
2. Alcohol Swab
3. Phlebotomy needle
4. Gauze
5. One 6-ml, labeled purple top EDTA blood collection tube (labeled with study ID number and date of birth)
6. Adhesive bandage
7. Ashion Specimen Requisition Form

## III. PROCEDURES

This procedure is intended to ensure that blood samples will be obtained from consented participants in a safe and efficient manner while eliminating the risks of contamination. Patient identifiers will remain under HIPAA compliance, and clinical information will be kept in accordance with each site’s standard practice. Sample collection kits containing the collection tube for shipping samples to the appropriate laboratory will be provided to the collection sites.

### A. Timing for Blood Collection

1. Preferably, if the participant will be undergoing surgery or biopsy, blood collection should be done pre-procedure and as close as possible to the time when the tissue is collected for the research study.

2. Identify the person responsible for processing the blood.

3. Contact this person before or soon after blood collection to arrange timely processing of the sample.

**B. Blood Collection Procedure – Preparation**

1. Blood collection must be performed by personnel qualified to draw blood.

2. Prior to blood collection, identify the participant, verify identification, and check that informed consent has been obtained.

3. All samples must be labeled with **2 patient identifiers** including the patient's study ID and Date of Birth. Both identifiers will be required in order for the CLIA laboratory to accept the sample.

4. Ensure that the labels on the blood collection tube match the participant study identification number on the informed consent document.

5. Assess participant's physical and mental disposition and determine if this is the appropriate time to draw blood.

6. Be courteous, professional, and sensitive to the participant's needs. Ensure that all communications are discreet and respectful of participant confidentiality.

7. Assemble proper equipment to draw blood.

**C. Blood Collection Procedure - Venipuncture**

1. Provide for the participant's comfort as much as possible, and gain the participant's cooperation. Position the participant. The participant should sit in a chair, lie down or sit up in bed. Hyperextend the participant's arm.

2. Apply tourniquet to expose veins. Do not place too tightly. If superficial veins are not easily apparent, force blood into the vein by massaging the arm from wrist to elbow, tap the site with index and second finger, apply a warm, damp cloth to the site or lower extremity to allow veins to fill.

3. Select appropriate site for venipuncture. Avoid areas with excessive scars or hematomas. While hand and wrist veins are acceptable it is optimal to select an antecubital vein.

4. Prepare the participant's arm using an alcohol prep. Cleanse in a circular fashion, beginning at the site and working outward. Allow to air dry.

5. Anchor the vein and swiftly insert the needle (at a 15-30 degree angle with the surface of the arm) into the lumen of the vein. Avoid excessive probing and trauma to the site.

6. Draw blood into one 6-ml purple top EDTA blood collection tube.

7. When the last tube is filling, remove the tourniquet.

8. Remove the needle from the participant and apply a gauze and adequate pressure to the site of venipuncture to avoid hematoma formation.

9. If needed, apply an adhesive bandage to the venipuncture site.

10. Dispose of needles and supplies in a safe manner.

11. Samples should be slowly inverted 8 to 10 times to ensure the mixing of the sample and the anti-coagulant liquid inside the tube.

12. Complete the Ashion Specimen Requisition form and include this form in the shipment (section D).

13. Ship immediately with a cool pack for overnight delivery with arrival Monday-Friday. Specimens can be refrigerated for 7 days before shipping if needed.

**D. Completion of the Ashion Sample Submission and Requisition Form**

**1.** Complete the Ashion Sample Submission and Requisition Form.

**2.** The patient's study ID number may be used (in place of name).

**3.** Please complete all required fields that are not pre-populated with study information.

**E. Shipping Blood to Ashion**

**NOTE: Ship Monday through Thursday only unless prior notification is made is made with Ashion. Do not ship the day before a U.S. Holiday.**

1. Verify the vacutainer label matches the patient study ID number and date of birth on the Ashion Specimen Requisition Form.

2. Place the blood vacutainer(s) in a leak-proof biohazard bag containing absorbent material.

3. Place frozen ice/cooler packs in the bottom of the provided box.. Place biohazard bag in the center of the cooler on top of the frozen ice/cooler packs, and then place another frozen ice/cooler pack(s) on top.

   place all paperwork associated with the case in the box, and tape shut.

4. The outermost container must be marked with the words Exempt Human Specimen (use labels or write by hand when necessary).

5. The U.S. DOT does not require these labels; however, IATA does require these labels. Therefore, include these labels on all packages in this category to streamline processes. Do not put the universal biohazard symbol on the outside of an exempt package as this may cause confusion regarding classification.

6. Verify that the shipping air bill is marked **Standard Overnight Shipping**

7. Call Courier Service to pick up specimens

8. Ship frozen tissue to:

    Ashion Analytics, LLC
    445 North 5th St. Suite 468
    Phoenix, AZ 85004
    602-343-8796
    602-343-8545 (Fax number)

9. Send an email to Ashion with the shipment date and tracking number at meahearn@ashiondx.com.

10. Questions to Ashion should be directed to the Laboratory Supervisor, **Mary Ellen Ahearn** (meahearn@ashiondx.com or 602-343-8659).

# Appendix 3: Fresh Frozen Tumor Collection and Shipment

The purpose of this Standard Operating Procedure (SOP) is to outline the process for snap freezing tissue for processing and storage in Ashion. Tissue samples will be collected from participants who have been properly consented and who have agreed to participate in the research study. Tumor tissues are only suitable for molecular studies if frozen in a timely and appropriate manner. The purpose of this document is to outline standardized procedures for collection sites to follow for snap freezing tissue.

## I. SAFETY

A. Wear personal protective equipment (PPE), such as lab coats and gloves when handling liquid nitrogen.

B. Liquid nitrogen is extremely cold and can cause ‘burns’. Wear gloves that are specially made to withstand liquid nitrogen, as well as eye protection and a lab coat to protect skin from splashes and spills.

## II. MATERIALS & EQUIPMENT

1. Container with dry ice (for transport of frozen tissue)
2. Clean forceps
3. Liquid nitrogen
4. 2-methylbutane (isopentane**)**
5. Container for isopentane
6. Labeled cryovials (with study ID number and date of birth)
7. Dry shipper
8. Shipping Airway Bill
9. Biohazardous bag for shipping
10. Dry Ice
11. Personal protective equipment (PPE) to include gloves, lab coat.
12. Ashion Specimen Requisition Form

## III. PROCEDURES

This procedure is intended to ensure that tissue samples collected from consented participants will be frozen in a safe and efficient manner while eliminating the risks of contamination and loss of molecular integrity. To facilitate the use of molecular techniques, tissue that has been adequately frozen is vital to obtaining products with high integrity and quality.

For each biopsy, we will collect four to six, 1-2 centimeter 18-gauge core needle specimens from accessible tumor. Depending on need (and as specified by the Principal Investigator for each patient enrolled on this study), either one or two cores

will be placed in pre-cooled cryovials and immediately flash frozen in liquid nitrogen and stored at -80$^{o}$C until shipping on dry ice to Ashion in Phoenix, Arizona.

### A. Snap freezing of Tumor Tissue

1. Label the cryovial. All samples must be labeled with **2 patient identifiers** including the patient's study ID and Date of Birth. Both identifiers will be required in order for the CLIA laboratory to accept the sample.

2. Treat all tissue as potentially infectious.

3. Freezing is performed by research study staff as designated by the collection site principal investigator.

4. Have materials and equipment ready for tissue processing prior to surgery. Have pre-labeled cryovials ready.

5. Fresh tumor tissue should be frozen as soon as possible. Optimally, tissue should be frozen within 30 minutes from biopsy.

6. Do not freeze the tissue directly on ice.

7. Ensure that the biopsied tissue never desiccates or is contaminated by surrounding tissue or other samples. Use clean forceps between samples to avoid cross contamination. Do not place the sample in contact with formalin at any point in the process. Do not add serum to the sample.

8. Cool isopentane by suspending the container of isopentane in liquid nitrogen.

9. Isopentane is sufficiently cooled when “pearls” form and the solution becomes hazy.

10. With clean forceps, place the specimen to be frozen into an empty screw capped cryovial.

11. Close the cryovial.

12. Place the cryovial with the specimen into the container of cooled isopentane.

13. The specimen should freeze within 30 seconds.

14. Alternatively, the isopentane freezing step can be optional. Place the tissue specimen into an empty cryovial, close the cryovial, and immediately submerge the cryovial into liquid nitrogen. The specimen should freeze within 30-60 seconds. This is not recommended if the sample is large in size, as longer freezing time will result in ruined morphology.

15. Once snap frozen, samples should be packaged with dry ice and immediately shipped to Ashion.
16. If samples cannot be immediately shipped, samples should be placed on dry ice to be carried to the freezer or liquid nitrogen storage facility for storage until shipping.
17. Complete the Ashion Specimen Requisition Form.
    a) Send the original form with the specimen.
    b) Keep a copy of the requisition in the patient's study binder if applicable.
18. **Submit a de-identified, coded copy of the pathology report with the sample.**

**B. Completion of the Ashion Sample Requisition Form**

1. Complete the Ashion Sample Requisition Form.
2. The patient's study ID number may be used (in place of name).
3. Please complete all required fields that are not pre-populated with study information.

**C. Fresh Frozen Tissue shipment to Ashion**

**NOTE: <u>Ship Monday through Thursday only unless prior notification is made is made with Ashion. Do not ship the day before a U.S. Holiday.</u>**

1. Verify the cryovial label matches the patient study ID number on the Ashion Specimen Requisition form.
2. Place cryovial containing frozen specimen in biohazard bag.
3. Place 2-3 inches of dry ice in the bottom of a Styrofoam cooler. Place biohazard bag in the center of the cooler on top of the dry ice, and then fill the cooler the rest of the way with dry ice (preferably pelleted). Place a single paper-towel or piece of paper across top of ice, then put lid on the cooler and tape the lid tightly to the cooler, sealing all the way around the lid.
4. Place the cooler in the cardboard box, placing all paperwork associated with the case on top of the cooler, and tape shut.
5. The outermost container must be marked with the words Exempt Human Specimen (use labels or write by hand when necessary).
6. The U.S. DOT does not require these labels; however, IATA does require these labels. Therefore, include these labels on all packages in

this category to streamline processes. Do not put the universal biohazard symbol on the outside of an exempt package as this may cause confusion regarding classification.

7. The outermost container must be labeled with a hazard class 9 label, UN1845, and net weight of dry ice in kilograms. The label should be affixed to a vertical side of the box (not the top or bottom) and orientated as shown in the picture in Appendix V. The maximum allowable net quantity of dry ice allowed per package is 200kg.

8. Verify the following on the shipping air bill:

    a) Standard Overnight Shipping

    b) The Airbill must include the statement “Dry ice, 9, UN1845, number of packages X net weight in kilograms”. FedEx has a check box on their Airbill to satisfy their requirement.

9. Call Courier Service to pick up specimens

10. Ship frozen tissue to:

    Ashion Analytics, LLC
    445 North 5th St. Suite 468
    Phoenix, AZ 85004
    602-343-8796
    602-343-8545 (Fax number)

11. Send an email to Ashion with the shipment date and tracking number at meahearn@ashiondx.com.

12. Questions to Ashion should be directed to the Laboratory Supervisor, **Mary Ellen Ahearn** (meahearn@ashiondx.com or 602-343-8659).

## Shipment Packing Instructions for Frozen Shipments

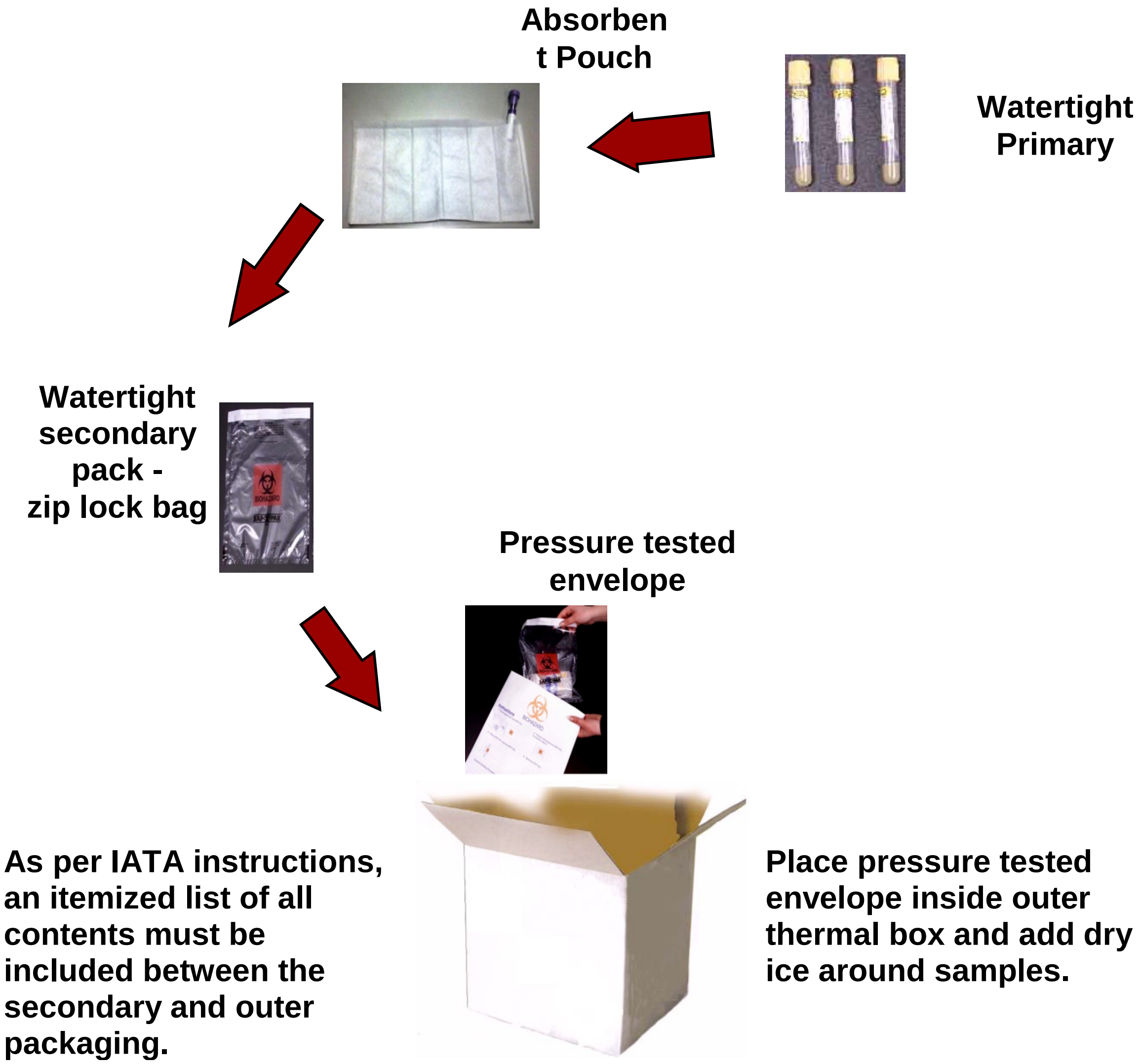


**Shipment Packaging Instructions for Frozen Specimen**
